# Interprofessional Education (IPE) and Pharmacy in the UK. A Study on IPE Activities across Different Schools of Pharmacy

**DOI:** 10.3390/pharmacy4040028

**Published:** 2016-09-26

**Authors:** Nilesh Patel, Shahmina Begum, Reem Kayyali

**Affiliations:** 1 Department of Pharmacy, University of Reading, Food Biosciences Building, PO Box 226, Whiteknights, Reading RG6 6AP, Berkshire, UK; 2 Department of Pharmacy, Kingston University, Penrhyn Road, Kingston upon Thames KT1 2EE, UK; shahe.begum@gmail.com (S.B.); R.Kayyali@kingston.ac.uk (R.K.)

**Keywords:** interprofessional education, interprofessional learning, pharmacy, healthcare professionals

## Abstract

Interprofessional education (IPE) has been recognised internationally as a way to improve healthcare professional interactions and team working in order to enhance patient care. Since pharmacists are increasingly part of multi-professional healthcare teams and are expanding their clinical roles, many pharmacy regulators have stipulated IPE must be included in educational curricula. This study aimed to examine how different Schools of Pharmacy (SOPs) in the UK implement IPE within their pharmacy course. Information about IPE was mainly obtained through interviews with staff from various SOPs. Nine telephone interviews were conducted which were analysed using a thematic analysis approach in order to derive common categories. These were identified as students, activities, barriers and facilitators and benefits of IPE. It was found that teaching methods used for IPE varied across SOPs. No standard strategy to deliver IPE was identified. Students were thought to value the IPE experience, especially the interaction with other professionals. The main barriers to implementing IPE arose from limited financial and organisational support. In general, many SOPs in the UK are undertaking IPE but challenges remain in establishing it as a routine part of the course, something which seems to echo difficulties in implementation of IPE both nationally and internationally.

## 1. Introduction

Internationally, the importance of healthcare professionals working together to create an optimal health care system has been recognised. The World Health Organisation (WHO) has noted that in order to better integrate care, strengthen quality and improve patient safety, interprofessional education (IPE) is necessary [1]. IPE is defined as occurring *“when two or more professions learn with, from and about each other to improve collaboration and the quality of care”* [2]. Therefore, the potential benefits of getting different healthcare professionals together to learn from each other and understand each other’s roles in order to improve patient care and safety have been a driver to implement IPE within professional curricula and practice.

In recent years it has become more evident that patient cases have become more complex and the inclusion of more than one profession in their care has therefore increased [3]. There are also increasing public reports about poor standards of patient care across healthcare sectors in the UK with suggestions that better team working and communication are needed amongst healthcare workers. IPE is a possible solution to some of these issues as interprofessional working has helped decrease medical errors and helped improve patient satisfaction, patient care and knowledge and skills of professionals [4]. Knowledge of different working practices and awareness of different professional accountabilities, roles and competencies are pivotal in driving improved healthcare [5,6]. Working together for patients requires teamwork and an appreciation of not only the types of services provided but of the providers themselves [7,8], which makes IPE highly relevant.

The development of the understanding of the roles and responsibilities of all health and social care professionals is undertaken at both undergraduate and postgraduate levels through various activities ranging from university-based classroom activities, forums and practice-based or workplace placements. In a recent review by Barr et al. [9] that looked at IPE in the UK over the period 1997–2013, it was reported that at least two-thirds of UK universities with qualifying courses in health and social care included IPE, outlining the growing importance of IPE. There is some suggestion that for IPE to have the biggest impact on healthcare professionals it should be incorporated early on in their education [10,11,12,13].

In the UK, the pharmacy regulatory body, the General Pharmaceutical Council (GPhC), makes it a requirement for Pharmacy (MPharm degree) courses to demonstrate “*learning based on experience that provides education in interprofessional practices and procedures with other healthcare professionals*” [14]. Nearly all MPharm courses in the UK are four years in length, and are commonly described as Levels 4–7, or Parts 1–4. It is not clearly stipulated by the GPhC at which level/part IPE should be undertaken; therefore, there is likely to be variation in when and how it is delivered. The requirement for Pharmacy courses to undertake IPE is not unique to the UK. In the United States (US), the learning outcomes in the Accreditation Council for Pharmacy Education (ACPE) Accreditation Standards and Guidelines were changed to include IPE and make it a priority, and in the most recent ACPE standards [15], IPE is now included as a standalone standard (Standard 11) and describes the key elements in IPE education as being team dynamics, team education and team practice. The importance of having IPE in pharmacy is not just restricted to the UK or the US but has also been included in Pharmacy curricula in Germany, Poland, Australia and elsewhere [15,16,17,18,19,20].

In addition, the education of healthcare professionals increasingly relies on demonstrating competency and mapping against competency frameworks. For IPE, a US collaborative, representing various healthcare courses including pharmacy, has created core competencies for interprofessional collaborative practice (IPEC) to guide curriculum development across health professions schools [21]. In the UK there is the Interprofessional Capability Framework, which has been developed to serve a similar purpose [22]. These curriculum frameworks provide a foundation for what students are expected to demonstrate in terms of knowledge, skills, values and attitudes. However, there are limitations in using these frameworks for IPE [23].

In a study by Jones et al. [24], it was noted that the delivery of IPE was not homogenous across pharmacy education programmes across the US, and that various barriers had to be overcome to implement IPE effectively. This may also be the case in the UK, but evidence is lacking as to how IPE is delivered and what the specific barriers for SOPs in the UK are. Therefore, an investigation into the engagement with IPE in UK SOPs was undertaken to achieve the following aims: to find out if IPE is undertaken by SOPs in the UK, discover the types of IPE activities which are undertaken and identify the barriers and benefits of IPE as perceived by pharmacy staff involved in IPE.

## 2. Methods

A purposive sampling technique was used to recruit participants for this exploratory study. The primary purpose of the study was to gather data about types of IPE activities undertaken within UK SOPs, and a secondary purpose of gathering data that would explore pharmacy staff experiences of IPE. Ultimately, it was hoped that a best practice model of IPE (i.e., a standard way of complying with IPE requirements) could be derived from the information obtained. A list of staff at 26 Schools of Pharmacy (SOP) in the United Kingdom (UK) was compiled. School of Pharmacy websites were used to collect contact details of staff that we believed to be leading or associated with IPE, or the Head of the SOP when this was not evident. Twenty-four SOPs were emailed a participant information sheet and an invitation letter outlining the study aims and an invitation to provide a reflective account of initiating and running IPE as well as what activities were undertaken. Two SOPs were not contacted as data has already been collected about their IPE involvement. A follow-up email was sent to the staff after two weeks. Invited staff either replied back with redirection to another staff member, agreed to participate (followed up with a convenient date and time for an interview) or declined to participate. No further follow-up occurred for the non-responders.

All participants who agreed to be interviewed gave their informed consent for inclusion before participating in the study. Interviews were conducted over the telephone (during March–April 2013) using a semi-structured guide, and the information recorded by hand by the researcher. The questions that were asked came under the general themes of IPE activities (for example, do they undertake IPE? what types of activities are undertaken? what topics are covered? is there an assessed component to IPE?), staff/student involvement (for example, which students are exposed to IPE? which staff are involved in teaching?), evaluation of IPE (for example, what feedback is received from students?) and what are the barriers and facilitators in undertaking IPE? Each interview lasted approximately 30 min. All information was anonymised with each interviewee given a code to prevent association to defined quotations. Transcriptions were analysed using a thematic analysis approach, which was carried out using the methodology of Ryan and Bernard [25]. Two researchers carried out the data analysis in various stages to reduce the possibility of any researcher bias during the category development. Categories were confirmed and verified by detailed line-by-line reading of the transcripts, which were further refined and reduced until a final list of categories were obtained and agreed by both researchers. At the end of the study the transcripts were destroyed. Ethical approval was granted by the Science, Engineering and Computing (SEC) Research Ethics Committee at Kingston University on 10 January 2013.

## 3. Results

Twenty-six SOPs were identified from which information about IPE was sought. However, only 34.6% (*n* = 9) of the contacted SOPs agreed to participate in a telephone interview. Three other SOPs had responded but declined to participate in an interview. One SOP reported that at the time of the study no IPE was undertaken. The written information provided by the two SOPs that did not participate in an interview was incorporated, when relevant, to the categories derived from the interviews.

Analysis of the data led to the derivation of four main categories: students, activities, barriers and facilitators and benefits of IPE.

### 3.1. Students

It was noted that five SOPs run IPE for all levels of the MPharm degree and three SOPs run it for only Level 7 of the MPharm. The rest of the SOPs provided IPE over a mixture of levels. Fifteen traditional healthcare courses (HCC) were involved in IPE across the investigated SOPs. These were medicine, nursing (including mental health, paediatric and adult), midwifery, physiotherapy, radiography (including diagnostics and therapeutic), paramedics, occupational therapists (OT), operating department practitioners (ODP), speech and language therapists, dietetics, dentistry, optometry, nutrition, podiatry and audiology. Medicine, nursing and midwifery appeared to be common HCCs to undertake IPE with. Non-healthcare courses (non-HCC) cited were psychology, occupational health, health science, health and social care, vision science, social work (commonly encountered), applied biomedical sciences, biology, police and youth and community work.

Participants recognised the importance and benefits of pharmacy students working with other courses not traditionally related to healthcare, for example social carers, whose profession had been criticised in one London Borough for not safeguarding against the abuse over an eight-month period of a 17-month-old child (“Baby P”). *“Working with non-healthcare courses would be beneficial as the pharmacist role is promoted and gives possible insight into patient perspective”* *(****Participant A****)*. *“Importance of social care is demonstrated by recent cases about safeguarding and childcare like Baby P. Social care is important when addressing issues about asylum seekers, vulnerable adults and children”* *(****Participant Y****). “Hospital scientists are commonly neglected, but when in clinical settings medical staff are dependent on them for test results. IPE allows appreciation of these non-healthcare courses” (**Participant X**)*.

When asked about what the students thought about working with other courses, a few participants mentioned that pharmacy students were more comfortable and confident when working with other healthcare students of a similar level and clinical experience, especially when it came to showing others what they could do as pharmacists. *“Students mixed in their preference; some prefer nurses due to their vast amount of clinical experience. Some prefer medics as they get to showcase their role as a pharmacist, due to medics underestimating the importance of a pharmacist in patient care” (**Participant A**). “I find pharmacy students prefer to work with students who have clinical experiences and do not like to work outside of the clinical setting” (**Participant Y**)*. *“Students felt scared and intimidated by the postgraduate medics and nurses due to their breadth of knowledge and experience. Doctors were more challenging whilst the nurses were ‘motherly’ or nurturing” (**Participant I**)*.

### 3.2. Activities

The majority of the SOPs run IPE activities once a year. Usually activities consume a full day unless students are sent on a placement. Many of the SOPs follow up IPE sessions with work for students to complete without the facilitator. The activities themselves are undertaken either on campus, or off-campus.

Activities hosted by the different SOPs on campus have some structural similarities to each other and tend to be lecture- or workshop-based. These different types of activities are outlined in Table 1.

With regards to off-campus events, many SOPs opt to send their students on placements (for example, hospitals, nursing homes, general practitioner (GP) practices) with other health and social care students. The students are required to complete activities related to their visit. Types of activities on placement include shadowing professionals for a day, observing interprofessional work in practice, going through case studies or ethical scenarios, and interacting with patients and/or carers.

Some SOPs summatively assess their IPE activities. For example, some components of the activity are assessed in an end-of-year exam, or there is a reflective assignment or poster presentation or a portfolio to complete. One SOP uses objective structured clinical examinations (OSCEs) to assess IPE.

### 3.3. Barriers and Facilitators

All interviewees mentioned that the UK pharmacy regulator (GPhC) requires SOPs to undertake IPE in order to be accredited, with one mentioning that these requirements are not the same for some of the other healthcare profession courses. IPE involvement was also thought to be hindered by the fact that not all universities and therefore SOPs have access to a wide range of healthcare courses, or there is competition with nearby SOPs. *“Differing standards and requirements for healthcare professional courses make the emphasis on IPE differ between the courses. Therefore, MPharm staff are more likely to try and incorporate IPE into their course due to the strict GPhC requirements, which regulate the course. Currently there are no requirements for nursing and medicine to incorporate IPE into their course. Also, these courses do not have strict regulations on their content as MPharm”* *(****Participant A****). “Unfortunately, cannot involve other healthcare professional courses as the degrees do not take place in the university and nearby universities have their own pharmacy courses. This leads to a considerable difference in IPE teaching” (**Participant Y**).* However, it should be noted that both the Nursing and Midwifery Council [26] and the General Medical Council [27] state that they expect students to engage in IPE during their education.

In addition, timetabling IPE events within the pharmacy course and with other healthcare courses was seen as particularly problematic. *“Both schools of teaching have hectic timetables, which make it difficult to organise formal slots in both timetables for IPE sessions”* *(****Participant A****)*. *“It’s a nightmare with another university. Especially trying to find time when medics and pharmacy students are free. Medics are taught on a rotation basis between September and July, whereas pharmacy students are taught between September and March” (**Participant S**).*

In terms of facilitating sessions, most SOPs stated that the pharmacy practice staff is mainly tasked with running all IPE activities, with often only one member of the staff responsible for the organisation. The presence or lack of an IPE organisation structure was seen to vary between SOPs and was cited for many as making organising IPE problematic when no clear structure was in place. Particular examples of where there seemed to be a formal organisation of IPE include one SOP that reported that they had teams dedicated to IPE, where an IPE lead overlooks all the teams for each year group. The team consists mainly of pharmacy practice staff and some non-pharmacy-related staff, medical practitioners and lecturers to show that interprofessionalism is practiced as well as taught. Similarly, another SOP had a steering group to strategise IPE, an organisation group that ensures day-to-day running which includes an IPE champion who communicates with the coordinators and ensures IPE achieves the objectives for the SOP, an IPE e-learning group that develops online IPE activities and an IPE research group that undertakes research and writes reports about IPE. One participant stressed the importance of having an *“expert”* administrative staff member who is able to organise the logistics of the day and the placements.

During the course of discussion around the area of IPE activities, comments about funding arose. Mainly, IPE was funded by the participating courses. It was of interest that sources of funding were sought by some SOPs. These included higher education grants, Health Education England (HEE) funding, supplementary funding from the government and one SOP was awarded £1000 from an internal funding bid. One SOP stated that their IPE was funded by a service-level agreement with collaborative hospitals whereas another discussed a future collaboration with the police force whereby the police force would fund the session under “*knowledge impact”* as part of the police knowledge fund.

### 3.4. Benefits of IPE

A uniform agreement amongst interviewees was that IPE is beneficial. Each SOP highlighted benefits that they felt their students gained from IPE during their time at university and after they had left. Some participants commented on improvement in skills and knowledge of their students when working with other healthcare professionals. *“...vital skills in people and mannerism are developed and improved during IPE sessions” (**Participant F**). “IPE provide students with an opportunity to increase clinical knowledge and ability to understand the social aspects of a patient” (**Participant S**)*.

Many SOPs stated that IPE has provided students with a better understanding of the roles of other professionals in patient care, not only the common ones they are likely to encounter. *“…allows medics to understand the role of a pharmacist” (**Participant W**). “…use knowledge and develop relationships for effective patient care. Break down barriers between professionals” (**Participant C**). “*…*allows interaction with under-represented professionals in patient care like Operating Department Practitioner”* (***Participant F****)*.

## 4. Discussion

There is little published data on IPE conducted within SOPs in the UK. Most of the studies examining pharmacy involvement in IPE are from the US and elsewhere, with much of the focus and perspectives being from the medical and nursing professions [28]. Therefore, this study is one of the first in the UK to provide a snapshot of IPE in some SOPs, with the degree to which IPE is seen to occur across the SOPs varying greatly. In an environment of multidisciplinary team work, knowledge about other professionals will ideally make it easier to redirect and correctly identify the professional needed to meet the needs of the patient. Findings from the study have shown that a large number of professions and non-healthcare professions have been involved in IPE. For some of these professions their importance to pharmacy may not be apparent, for example the police, biomedical science students and social workers. However, healthcare is quite complex and involves many different people and so students should at least be aware of the roles of not only those people directly involved in the care of patients (medics and nurses, which some participants suggested their students were wanting to be more engaged with) but some of the others mentioned above. In addition, for those participants who mentioned difficulties in setting up IPE due to lack of available professions, thinking more widely of other people involved in the care of people with healthcare needs may be of benefit (as one or two SOPs have already done).

Generally, many of the participants felt students were gaining an understanding of their role and the role of other professionals in patient care. Indeed, a recent literature review undertaken by The Royal College of Nursing found that IPE enabled students to have a positive attitude and perception of other professionals’ contribution to a patient care pathway [29]. A report on new medical, nursing and pharmacy graduates’ reflections on their experiences of IPE during their undergraduate degree found that they valued IPE and regarded their experiences as positive [30]. Also, from our own study, IPE was thought to give pharmacy students an opportunity to promote their role as a pharmacist, which was thought to be under-rated by other professionals.

A common feature seen for many of the SOPs is exposing the students to IPE for one academic year only and usually in the early years, although some did prioritise IPE to final-year students (Level 7). Those SOPs that included IPE in the early years of the course tended to cover team working skills and learning about the roles of other professionals through ice-breaker sessions. Having IPE only in the final year may have been because these students are considered mature and better equipped in terms of clinical knowledge to be able to effectively interact with other healthcare professionals. However, IPE was rarely undertaken across all years, which some participants said was because of difficulties in finding appropriate professional partners and timetabling of the events. A couple of SOPs do seem to have overcome these barriers either because of the organisational support they receive or their close vicinity to other professional courses.

The type of IPE activities undertaken by SOPs also vary considerably (Table 1). These range from what might be considered as multiprofessional (for example, just having students from different professions sitting together in a lecture theatre) to truly interprofessional (by working very closely and interacting with other professions). Use of patient/case scenarios was common across all SOPs and this seemed to allow a variation of topics to be covered, although the main theme seemed to be around patient safety. Other examples of activities included buddy groups, conferences and placements. Peer learning was also mentioned, something which has been used with success elsewhere with physical therapy students teaching pharmacy students about ambulatory devices [31].

Some of these IPE activities are not dissimilar to those done elsewhere. For example, Odegard et al. [32] examined IPE initiatives undertaken at the University of Washington, which included introductory seminars, lecture-based courses, student-operated clinics, and an interprofessional objective structured clinical evaluation (OSCE). MacDonnell et al. [33] reported on pharmacy, medical and nursing students working together to diagnose and treat patients, whilst Rotz et al. [34] reported on having pharmacy and medical students participate in an interprofessional experiential course series. Activities not highlighted in our study, but which could be of interest to pharmacy courses, are the use of interprofessional training wards [35], or e-learning, the latter of which, when the study was undertaken, would have been less used within universities, although it is not without its challenges for being implemented in IPE [9,36].

There has been a recent increased emphasis on patient and public involvement in both teaching and research; therefore, one could argue that an important criterion for effective IPE is involving patients and clients in the design, teaching, participation and assessment of programmes [37,38]. One SOP set up an IPE conference day where patients, carers and service users were able to tell students their story. The service users were given a unique insight into the workings of a healthcare team when deciding on their care pathway and healthcare students were given a chance to understand the importance of involving the patient as much as possible in the treatment plan.

Some SOPs included a form of assessment of IPE, but we were unable to determine the rationale for why they did this. However, one possible reason would be to ensure student engagement with IPE. Indeed, Barr et al. [9] reported that students valued IPE more when it was assessed and that in the absence of some form of summative assessment IPE was given a lower priority by students, and interestingly, also by teachers.

What is evident across the different SOPs is that there is no standardised way in delivering IPE, despite there being a capability framework to follow. A conceptual framework does exist for developing a strategic plan for IPE (the Leicester Model), which has been adopted in various settings [39,40,41]. As one participant suggested, it is useful to have a dedicated IPE lead, a strategy group, and engagement from all staff, not only the pharmacy practice team, in order for IPE to be easy to deliver. Indeed, Barr et al. [9] also mentioned that having an IPE coordinator was important for the alignment of timetables and other logistical issues along with backing from line managers and institutional endorsement. This type of thinking is also echoed by Brazeau [42], who reported that in order to develop an effective IPE programme, investment in terms of time and money is needed, as well as a top-down administrative support and leadership approach. Thus, university support would be instrumental in overcoming some of these barriers.

The general lack of investment in interprofessional research and in evidence regarding the effects of IPE may compound this issue of organisational support further. However, it is noteworthy to mention a systematic review on the effectiveness of IPE by Reeves et al. [43], which states that there has been some useful progress being made in relation to strengthening the evidence base for IPE, but in order to provide a greater clarity of IPE and its effects on professional practice and patient/client care, rigorous mixed method studies of IPE are required to be undertaken.

Limitations to this study include that the interviews were not audio recorded and some of the information was derived from written documents. In addition, we were unable to ascertain if data saturation was achieved, partly as we were unable to speak to all the SOPs or complete a full thematic analysis. The low response rate may be because the study was undertaken during the academic term when the staff is busy teaching, or that there was more than one SOP at the time the study was undertaken that did not provide IPE. Information provided by the interviewees on the benefits of IPE was anecdotal and not based on acquired evidence. This does highlight that more robust and rigorous research is required to draw conclusions on the effects of IPE on professional work and its impact on patients, as currently these are limited and provide only evidence to support the positive outcomes of IPE events [29,44,45]. However, the current study does provide a snapshot of what IPE was undertaken at the time the study was undertaken.

## 5. Conclusions

There is a range of activities which are being used for IPE within UK SOPs, which this study is one of the first to explore. None of the UK SOPs have a common standardised approach to IPE which makes it difficult to compare and contrast IPE practices and define a best practice model of IPE. For many SOPs, if IPE is to be beneficial, certain barriers need to be overcome and there are lessons to be learnt from looking at good IPE practices seen within the UK and also internationally. Further research is needed to evaluate how IPE is undertaken and perceived by pharmacy students in the UK and the effectiveness of IPE on promoting better and safer work practices.

## Figures and Tables

**Table 1:** IPE activities undertaken by SOPs.

Type of Activity	Description of Activity
Teamwork and personality traits	Many SOPs mentioned that they get students to examine their personality type using Myers-Briggs Type Indicator (MBTI) tests, their role in a team and factors which can affect team performance using Belbin Team Roles as a way to promote the idea of team working. Typically this is done during an “ice-breaking“ session when the different healthcare profession students first meet.
Practice-based scenarios	In all cases there is some practice-based scenario involved in IPE. Students are typically divided into multidisciplinary groups to discuss a presented case or scenario and feedback to facilitators or professionals that may be involved in the case/scenario. The cases/scenarios have been on social care issues, professionalism, ethical dilemmas, health promotion, compromised patient safety, critical situation, and discharge meeting notes.
IPE days/conferences	A mixture of lectures and workshops occurring over a whole day. Generally, the day is based on a particular topic or disease. For example, the start of the day usually includes an introduction to the topic (e.g alcohol misuse, safeguarding children and vulnerable adults, drug charts, transfer of care and evidence-based medicines for prescribing were mentioned) with a video and brief lectures from healthcare professionals, patients or relevant organisations. These were then typically followed by mixed professional group workshops in the afternoon.
Peer teaching	This was mentioned by two SOPs whereby students from one profession teach other healthcare courses on topics within their specialisation. For example, in one SOP, fourth-year students teach physiotherapy students about the safe and effective use of medicines. In turn, they are taught by physiotherapy students about physical therapies. In another SOP, their students were taught by medicine and nursing students who themselves were trained in basic life support.
Buddy systems	In one SOP, first-year MPharm students are introduced to other first-year HCC students and are placed in mixed groups to complete tasks. During their time at university they are expected to remain within their group and organise their own meetings to undertake various tasks given to them, which has the added benefit of eliminating any timetabling problems.
